# A predictive nomogram improved diagnostic accuracy and interobserver agreement of perirectal lymph nodes metastases in rectal cancer

**DOI:** 10.18632/oncotarget.7548

**Published:** 2016-02-21

**Authors:** Yongfeng Liu, Renjie Wang, Ying Ding, Shanshan Tu, Yi Liu, Youcun Qian, Linghui Xu, Tong Tong, Sanjun Cai, Junjie Peng

**Affiliations:** ^1^ Institute of Health Sciences, Shanghai Jiao Tong University School of Medicine (SJTUSM) and Shanghai Institutes for Biological Sciences (SIBS), Chinese Academy of Sciences (CAS), Shanghai, China; ^2^ Department of Colorectal Surgery, Fudan University Shanghai Cancer Center, Shanghai, China; ^3^ Department of Oncology, Shanghai Medical College, Fudan University, Shanghai, China; ^4^ Department of Biostatistics, University of Pittsburgh, Pittsburgh, PA, USA; ^5^ Department of Statistics, Ohio State University, Columbus, OH, USA; ^6^ Department of Radiology, Fudan University Shanghai Cancer Center, Shanghai, China

**Keywords:** nomogram, rectal cancer, lymph node, metastases, MRI

## Abstract

**Objective:**

To develop a predictive nomogram to improve the diagnostic accuracy and interobserver agreement of pre-therapeutic lymph nodes metastases in patients with rectal cancer.

**Materials and Methods:**

An institutional database of 411 patients with rectal cancer was used to develop a nomogram to predict perirectal lymph nodes metastases. Patients' clinicopathological and MRI-assessed imaging variables were included in the multivariate logistic regression analysis. The model was externally validated and the performance was assessed by area under curve (AUC) of the receiver operator characteristics (ROC) curves. The interobserver agreement was measured between two independent radiologists.

**Results:**

The diagnostic accuracy of the conventional MRI-assessed cN stage was 68%; 14.2% of the patients were over-staged and 17.8% of the patients were under-staged. A total of 35.1% of the patients had disagreed diagnosis for the cN stage between the two radiologists, with a kappa value of 0.295. A nomogram for predicting pathological lymph nodes metastases was successfully developed, with an AUC of 0.78 on the training data and 0.71 on the validation data. The predictors included in the nomogram were MRI cT stage, CRM involvement, preoperative CEA, tumor grade and lymph node size category. This nomogram yielded improved prediction in cN stage than the conventional MRI-based assessment.

**Conclusions:**

By incorporating clinicopathological and MRI imaging features, we established a nomogram that improved the diagnostic accuracy and remarkably minimized the interobserver disagreement in predicting lymph nodes metastases in rectal cancers.

## INTRODUCTION

The goal for the treatment of rectal carcinoma is cure or local control of the disease with maintenance of an acceptable quality of life. As progresses achieved in the last two decades, total mesorectal excision and combined modality therapy, especially the preoperative chemoradiotherapy (CRT), has been the standard therapy for locally advanced rectal cancer. Compared to the postoperative CRT, preoperative CRT has demonstrated improvement in patient's local control and quality of life. However, the radiation or CRT related toxicities are still not insignificant; a substantial incidence rate of impaired short-term or long-term quality of life, including fecal incontinence and erectile dysfunction, has been reported in patients with preoperative radiotherapy or CRT [1–3]. Improving the accuracy of preoperative staging system can potentially help identify patients who may be optimal candidates to undergo preoperative CRT.

Currently, magnetic resonance imaging (MRI) is one of the best preoperative staging techniques, and is especially preferred in locally advanced rectal cancer by its assessment of T category, N category and circumferential resection margin (CRM) [4, 5]. However, although the preoperative MRI was proven to predict both T category and circumferential resection margin with good accuracy, the accuracy for predicting lymph node metastasis was poor [6].

Previous studies also found clinicopathological features, such as T category, lymphovascular invasion or tumor grade, which may help identify patients at high risk of lymph node metastases [7, 8]. However, very few study established a reliable prediction model, using both imaging variables and clinicopathological variables, to predict pre-therapy lymph nodes metastases. Furthermore, although very small lymph nodes can be observed by high resolution MRI, the diagnosis of metastasis from detected lymph nodes is challenging, and the interobserver agreement in diagnosis of lymph nodes metastases varies among radiologists, especially for sub-centimeter lymph nodes [9, 10].

Therefore, the objective of the current study was to develop a predictive nomogram to improve the diagnostic accuracy and interobserver agreement of pre-therapy perirectal lymph nodes metastases in patients with rectal cancer, by incorporating pre-therapeutic clinicopathological and MRI imaging variables.

## RESULTS

### Clinicopathological and conventional MRI characteristics

The clinicopathological characteristics of all the 411 patients were listed in Table 1, which were comparable between the training group and the validation group. The pathological stage I, II and III (AJCC staging, sixth edition [11]) patients were 28.5%, 28.7% and 42.8%, respectively. The median number of sampled lymph nodes was 13 (range, 4–58) in the training group and 14 (range, 6–49) in the validation group. Of the 411 patients, lymph nodes were not found by MRI assessment in 130 patients (31.6%), of which 26 (20%) out of 130 patients proved node positive by histopathology.

**Table 1:** The clinicopathological characteristics of 411 patients with rectal cancer

		Training Group		Validation Group		*P* value
		No. of patients(*N* = 288)	%	No. of patients(*N* = 123)	%	
Age	< /= 60	159	55.2	65	52.8	0.66
	> 61	129	44.8	58	47.2	
Gender	Male	163	56.6	71	57.7	0.833
	Female	125	43.4	52	42.3	
Tumor location*	< 5 cm from AV	163	56.6	78	63.4	0.0002
	5–10 cm from AV	88	30.6	45	36.6	
	> 10 cm from AV	37	12.8	0	0	
Preoperative CEA	< 5 ng/ul	205	71.2	82	66.7	0.361
	≥ 5 ng/ul	83	28.8	41	33.3	
Types of Surgery	Anterior resection	203	70.5	91	74.0	0.527
	Abdominal perineal resection	62	21.5	24	19.5	
	Hartmann's resection	23	8.0	8	6.5	
Tumor grade	Low-medium grade	249	86.5	102	82.9	0.353
	High grade	39	13.5	21	17.1	
MRI cT stage	mrT1-2	86	29.9	43	35	0.213
	mrT3	195	67.7	74	60.2	
	mrT4	7	2.4	6	4.9	
MRI cN stage	mrN−	176	61.1	63	51.2	0.063
	mrN+	112	38.9	60	48.8	
pT stage	pT1-2	95	33	47	38.2	0.308
	pT3-4	193	67	76	61.8	
pN stage	pN0	166	57.6	69	56.1	0.793
	pN1	76	26.4	31	25.2	
	pN2	46	16	23	18.7	
TNM stage, 6th ed.	stage I	79	27.4	39	31.7	0.444
	stage II	87	30.2	30	24.4	
	stage III	122	42.4	54	43.9	

Abbreviation: AV, anal verge; CEA, carcinoembryonic antigen; * The tumor location was measured endoscopically.

Of 288 patients in the training group, the detailed MRI imaging characteristics were studied and presented in Table 2. There were a total of 808 lymph nodes detected by MRI, in which 478 nodes (59.2%) were size < 5 mm, 310 nodes were size 5 mm–10 mm (38.4%), and 20 nodes (2.4%) were size > 10 mm. The mean number of detected lymph nodes was 2.8, ranging from 0–27.

**Table 2:** Univariate analysis of relationship between clinicopathological/imaging variables and pathological *N* stage

		pN (−)		pN (+)		*P* value
		No. of patients(*N* = 166)	%	No. of patients(*N* = 122)	%	
Gender	Male	94	57.7	69	42.3	0.991
	Female	72	57.6	53	42.4	
Age	≤ 60	97	61	62	39	0.199
	> 60	69	53.5	60	46.5	
Tumor location	< 5 cm from AV*	92	56.4	71	43.6	0.634
	5–10 cm from AV	50	56.8	38	43.2	
	> 10 cm from AV	24	64.9	13	35.1	
Preoperative CEA	< 5 ng/ul	128	62.4	77	37.6	0.01
	≥ 5 ng/ul	38	45.8	45	54.2	
Tumor grade	Low-medium grade	153	61.4	96	38.6	0.0017
	High grade	13	33.3	26	66.7	
MRI cT stage	cT1-2	66	83.5	13	16.5	< 0.00001
	cT3-4	100	47.8	109	52.2	
MRI CRM involvement	Clear	151	60.4	99	39.6	0.024
	Involved	15	39.5	23	60.5	
Tumor spectrum	< 1/2 circle of bowel wall	70	63.1	41	36.9	0.14
	≥ 1/2 circle of bowel wall	96	54.2	81	45.8	
Peritoneal reflex relationship	Above	35	55.6	28	44.4	0.705
	Below	131	58.2	94	41.8	
LNs size category	No LN detected	76	80.9	18	19.1	< 0.00001
	Largest LN size < 5 mm	41	58.6	29	41.4	
	Largest LN size 5–10 mm	48	44	61	56	
	Largest LN size > 10 mm	1	6.7	14	93.3	
Irregularity of nodes border	Detected	45	40.5	66	59.5	0.00003
	Undetected	121	68.4	56	31.6	
Uniformity of signal intensity	Detected	54	41.5	76	58.5	0.00001
	Undetected	112	70.9	46	29.1	

Abbreviation: AV, anal verge; CEA, carcinoembryonic antigen; CRM, circumferential resection margin; LN,lymph node.

The associations between MRI-assessed cN stage and pathological N stage (pN stage) were assessed in the training group. The diagnostic accuracy of MRI-assessed cN stage was 68%; the sensitivity, specificity, positive predictive value (PPV) and negative predictive value (NPV) were 58.2%, 75.3%, 63.4% and 71%, respectively. 14.2% of patients were overstaged and 17.8% of patients were understaged. While for the T stage, the diagnostic accuracy of MRI-assessed cT stage was 81.3% with12.2% of patients being overstaged and 6.5% of patients being understaged.

### Interobserver agreement of conventional MRI

**Figure 1:**
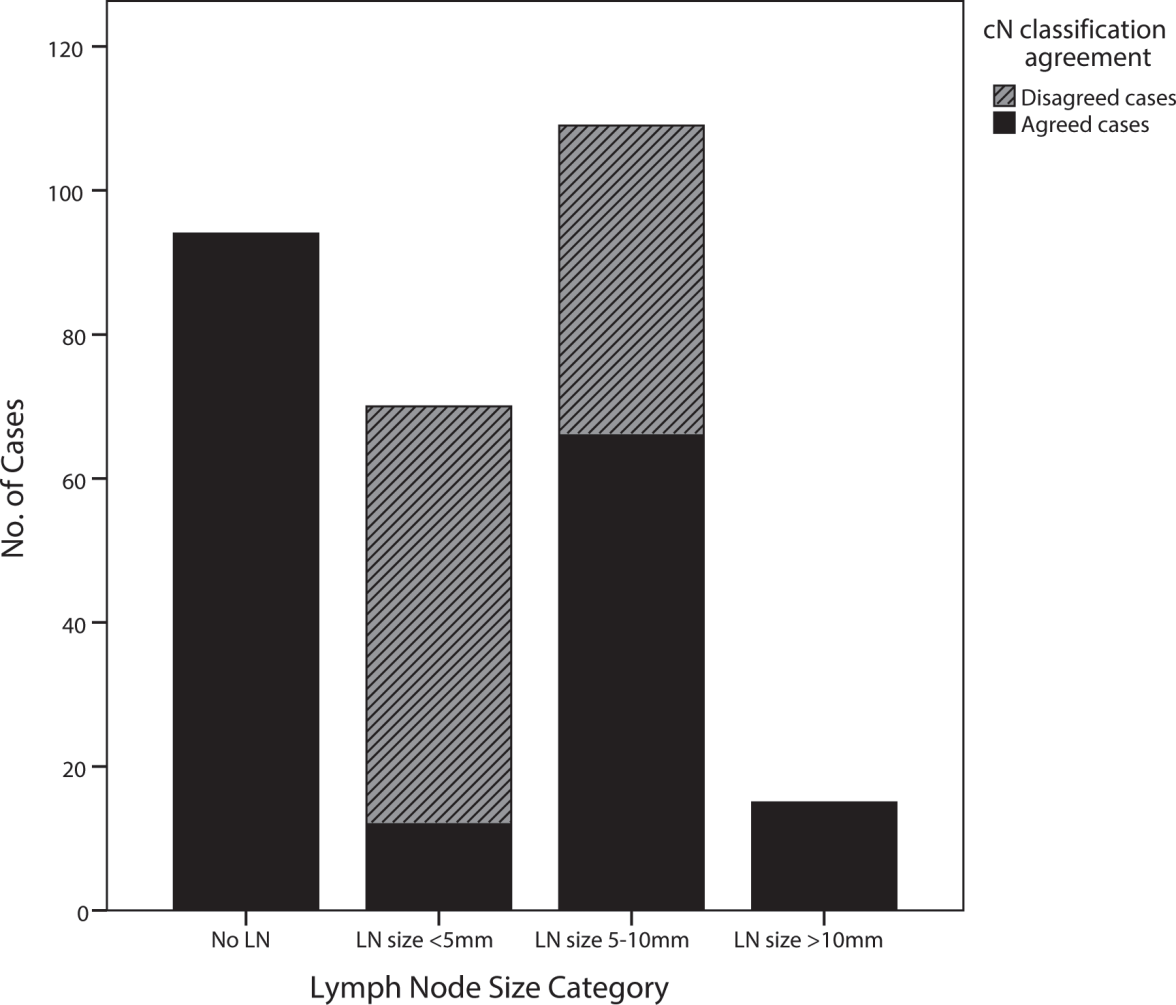
The comparison of diagnostic agreement of cN stage in MRI-assessed lymph nodes, categorized by their sizes (Black bars and grey bars represent agreed cases and disagreed cases by two radiologists, respectively.)

Of 288 patients in the training group, the overall diagnostic accuracy of cN stage was comparable between the two radiologists (65.6% for Doc 1 and 68.1% for Doc 2). However, for a case-to-case comparison, 35.1% of patients (101/288 cases) were disagreed between the two radiologists, with a kappa value of 0.295. Further investigation found that patients with undetected lymph nodes or with lymph nodes ≥ 10 mm yielded complete agreement of diagnoses between Doc1 and Doc2, while patients with disagreed diagnoses were those with small sub-centimeter lymph nodes. Of these 101 patients with disagreed diagnoses, 57.4% of cases had lymph nodes sized < 5 mm and 42.6% of cases had lymph nodes sized 5–10 mm (Figure 1).

### Nomogram development and performance

**Figure 2:**
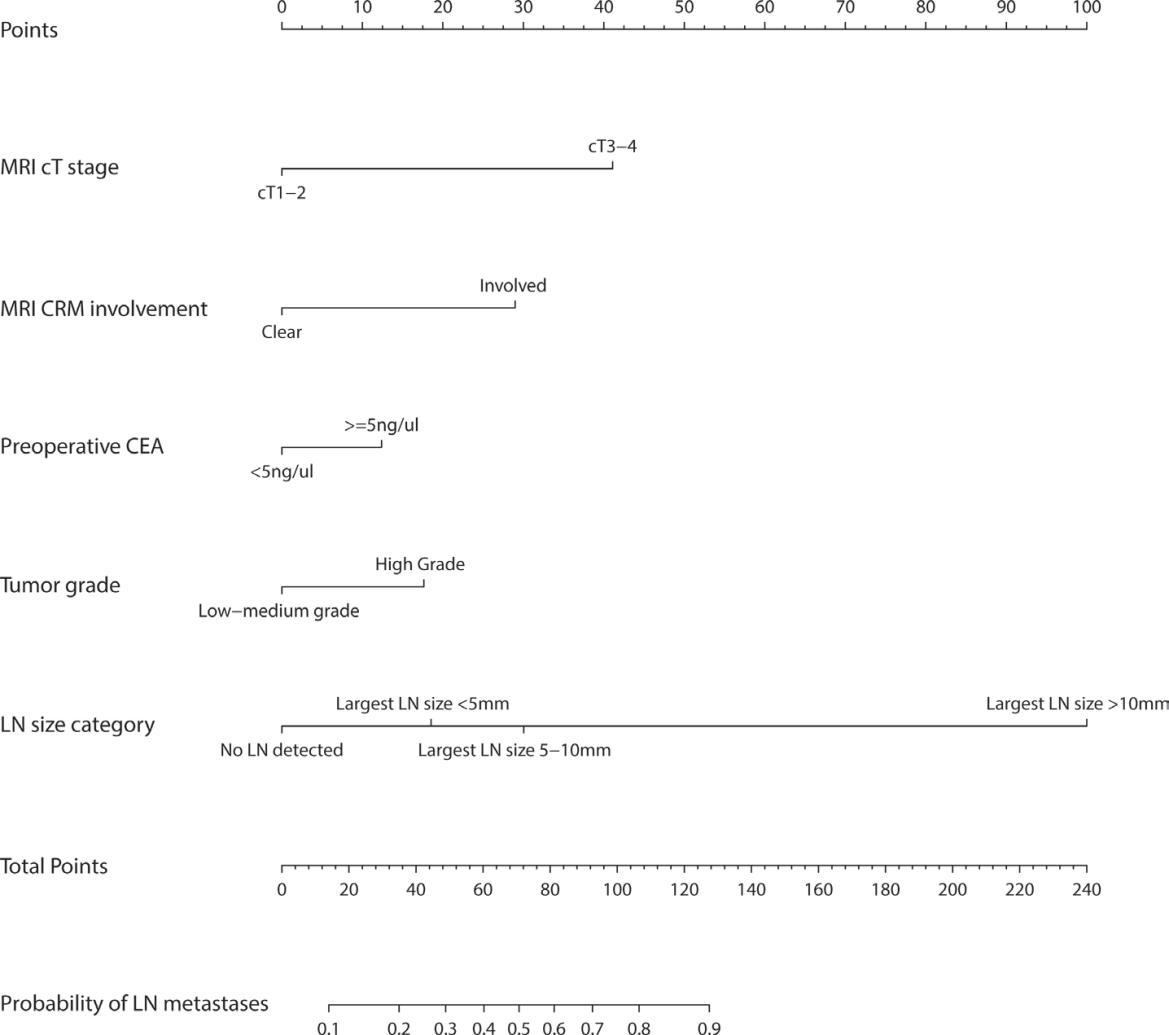
Rectal cancer nomogram for predicting lymph node metastases Each variable value is assigned a score, and the sum of scores is converted to a probability of pathological lymph node metastasis in the lowest scale.

The univariate analysis result of the clinicopathological and MRI imaging variables was presented in Table 2. For the ease of clinical application, the lymph node size category in our nomogram was classified into four groups using the largest size of detected lymph nodes within each patient: no LN detected, largest LN size < 5 mm, largest LN size 5–10 mm, largest LN size > 10 mm. The variables with a *p*-value < 0.1 were included in the multivariate analysis. The final nomogram was provided in Figure 2, with an AUC of 0.78 for the training data and 0.71 for the validation data. The predictors included in the nomogram were MRI cT stage, MRI CRM involvement, preoperative CEA, tumor grade and lymph node size category. From Figure 2, we can get a predicted probability of N stage positive for a given subject in the following way. First, use the top “points” line to get a point for each predictor (according to its value). Next, sum up the points from all predictors to get the total point. Last, map the total point to the probability line (i.e., “Probability of N+”) to get the predicted probability. For example, if the total point is 100, then the predicted probability of N positive is 0.75. Table 3 presents the odds ratio (OR) with 95% CI and the *p* value for each predictor.

**Table 3:** Multivariate analyses of predicting pathological lymph nodes metastases: the final predictor for developing the nomogram

Variable	Logistic Regression			Nomogram	
	Training Group (*N* = 288)				
	OR	95% CI	*p*-value	AUCs	95% CI
MRI cT stage					
cT3-4 vs cT1-2	4.91	[2.26,10.68]	5.87 * 10^−5^		
MRI CRM involvement					
Involved vs clear	3.07	[1.28, 7.40]	0.01		
Preoperative CEA				Training: 0.78	[0.732, 0.837]
≥ 5 ng/ul vs < 5 ng/ul	1.62	[0.88, 2.95]	0.12	Validation:0.71	[0.619,0.801]
Tumor grade					
High grade vs low-medium grade	4.91	[0.89, 4.42]	0.10		
LN size category					
largest LN size < 5 mm vs no LN detected	2.05	[0.95, 4.40]	0.07		
largest LN size 5–10 mm vs no LN detected	3.20	[1.60, 6.42]	1.04 * 10^−3^		
largest LN size > 10 mm vs no LN detected	48.09	[5.58, 414.34]	4.24 * 10^−4^		

Abbreviations: MRI, magnetic resonance image; CRM, circumferential resection margin; OR, odds ratio; CI, confidence interval; AUC, area under ROC curve; CEA, carcinoembryonic antigen; LN, lymph node.

**Figure 3:**
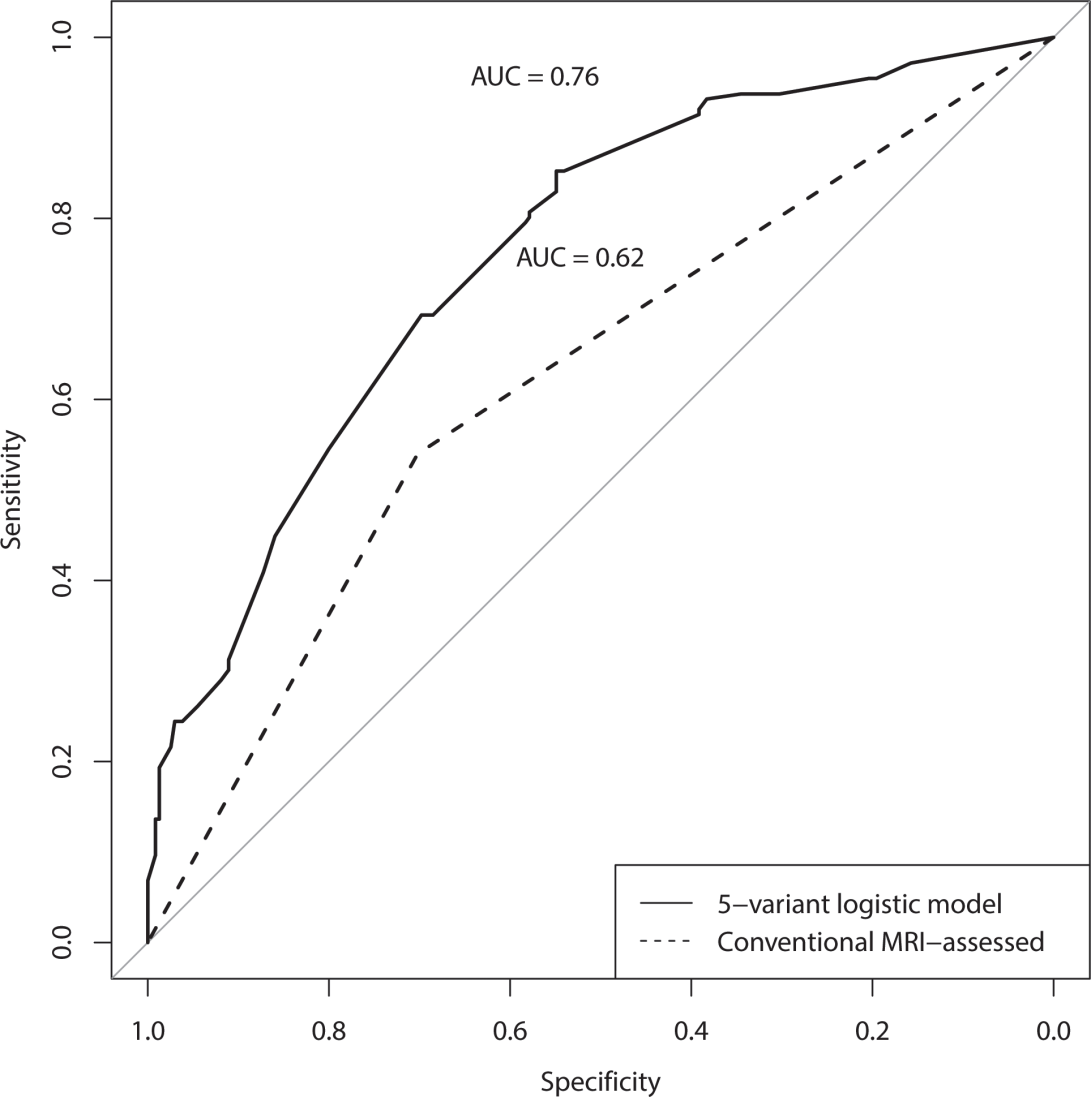
The ROC curves of the developed nomogarm and conventional MRI-assessed cN stage based on all patients

To compare the prediction performance, the ROC curves based on all 411 patients were plotted for both the nomogram and conventional MRI-assessed cN stage (Figure 3). The AUCs of the nomogram and conventional MRI-assessed cN stage were 0.761 (95% CI, 0.715–0.807) and 0.621 (95% CI, 0.574–0.668), respectively. Therefore, our nomogram was superior to the conventional MRI-assessed cN stage in predicting pathological lymph node metastases.

## DISCUSSION

In the current study with a large number of patients, we successfully developed a nomogram for predicting lymph node metastases in rectal cancers, by including both clinicopathological risk factors and magnetic resonance imaging features. Our nomogram showed improved diagnostic accuracy and could eliminate interobserver variability in the assessment of lymph nodes. Although lymph node metastasis is not the only criterion for identifying patients to undergo preoperative treatment, improved preoperative staging will reduce overtreatment or undertreatment, and ultimately improve patient's outcome and quality of life. Meanwhile, by informing patients the likelihood of lymph node metastases, patients can potentially become more actively involved in the decision-making process with regard to preoperative treatment with their doctors. It will also provide additional support to improve patient's treatment compliance.

Lymph node metastasis is a key indication for preoperative CRT and is an important prognostic factor for local or distant recurrence, which has been greatly emphasized in various treatment guidelines for rectal cancer [12, 13]. Consequently, the precision of treatment in patients with rectal cancer will be compromised by the large amount of patients with overstaged or understaged disease. In a specific series, 22% of patients still had lymph node metastases even after preoperative CRT, which were all diagnosed cT3 and node negative before treatment [14]. Although the authors stated that potential overtreatment was warranted in cT3N0 patients due to unsatisfactory preoperative node staging, a considerable number of patients may benefit from improved preoperative staging methods and obviate from the radiotherapy. Several meta-analyses have investigated the diagnostic accuracy of MRI for local stage of rectal cancer, including T category, lymph node metastases and CRM [4–6, 15]. The performance of MRI in diagnosing lymph node metastases was consistently poor, with varied sensitivity (60-84%) and specificity (59–81%) [6, 16]. In our series, using the conventional clinical staging, the overall diagnostic accuracy, sensitivity and specificity were similar to these previous studies, and both the percentages of overstaged cases and understaged cases were as high as around 15%.

One limitation in detecting positive lymph nodes by MRI is that a great number of detected nodes turned out to be normal or benign reactive nodes. In a node-to-node study of lymph nodes in rectal cancer, only 76 out of 521 nodes (14.6%) were confirmed malignant nodes by histology [17]. Currently, identifying malignant nodes mainly relies on the size of nodes. Studies confirmed that the proportion of positive nodes increased with larger nodal size [18–20]. However, there was no widely accepted consensus on the size criterion of enlarged lymph nodes, and substantial overlap exists between benign and malignant nodes. Brown et al. studied 476 lymph nodes in 42 patients with rectal cancers. In their study, although a cutoff of 5 mm gave optimal sensitivity and specificity for nodal status, 58% of positive nodes were less than 5 mm in diameter, and the overall predictive value by size was poor [20]. Similarly, 41.4% of patients in our series, who only had lymph nodes < 5 mm, were found node positive by histology. Other imaging parameters in MRI, such as border, shape or intrinsic signal of lymph nodes, were concurrently analyzed in various studies. However, the results were inconsistent. Kim et al. found that besides nodal size, lymph nodes with spiculated or indistinct border and mottled heterogeneous appearance were useful to predict positive nodes [19]. Brown et al. also found higher accuracy was achieved when lymph nodes with irregular border and mixed intrinsic signal were considered malignant [20]. However, in another study, even the criteria of positive lymph nodes were strict (heterogeneous texture, irregular margin, and nodal size > 10 mm), the total accuracy was only 63%, with a sensitivity of 85% and specificity of 41% [21]. Most of these studies only had a small number of patients and multivariate analyses were rarely used to assess the relationship between these parameters and the nodal size. Besides, in our point of view, determining the border, shape or intrinsic signal is difficult and inaccurate for very small nodes (less than 3–5 mm) in MRI. Some low-signal-intensity rim in small lymph nodes is difficult to distinguish between normal lymph node capsule and tumor with necrosis, and tumor without necrosis in lymph node will present similar intensity with normal lymph nodes. In our study, the border and intrinsic signal of nodes were also found significantly associated with node metastases, but both of them were excluded in the multivariate analysis due to the reason that 59.2% of detected nodes were less than 5 mm in our series. Although not optimal, the size of lymph node is currently the most reliable parameter for diagnosing lymph node metastasis in rectal cancer.

Clinical or histopathological features have been proved to be related to lymph node metastases in patients with colorectal cancer [22–25]. In early rectal cancer, some features, such as lymphovascular invasion, tumor size and T category, were used as risk factors to select candidates undergoing local excision without lymphadenectomy. Nevertheless, only a small number of studies focused on rectal cancer, and the examined clinicopathological features manifested varied significance in different studies [26]. Currently, no single clinicopathological feature of rectal cancer could optimally predict lymph node metastases. Using clinicopathological risk factors, a multivariate logistic model was established by Ogawa et al. which improved diagnostic accuracy of lymph node metastases by MRI [27]. However, the complicated criteria of MRI nodal interpretation and lack of external validation in that study restricted its practical use in clinical situations. In our study, with a large number of cases, a reliable and simple nomogram was successfully developed with external validation, based on variables from MRI and preoperative clinicopathological features. Compared with the standard MRI diagnosis, a significant improvement in predicting lymph node metastases was achieved by our nomogram.

The interobserver agreement was regularly examined in many studies of radiological lymph node assessment, and the results varied with different MRI techniques and study designs. The interobserver agreement was relatively higher in studies with node-to-node matched analysis than that with patient-to-patient analysis [28–30]. However, bias existed in both types of studies. Node-to-node matched studies could not assess cases with invisible nodes in MRI, in which a considerable number of patients still had lymph node metastases; meanwhile, patient-to-patient studies could not clearly ascertain that the suspicious nodes in MRI can be sampled and assessed by pathologists. These reasons may attribute to the variability of interobserver agreement. In our study, even with similar diagnostic accuracy, the interobserver agreement between the two radiologists was only fair for lymph node assessment, as compared to the excellent interobserver agreement for T category assessment. In addition, all the disagreed cases were located in patients with sub-centimeter lymph nodes, and patients only with lymph nodes less than 5 mm had greatest disagreement in the diagnosis of node metastasis. Our nomogram consisted of preoperative collectable clinicopathological variables and simple MRI node categories, where different scores, representing different weights in predicting lymph node metastases, were assigned to these predictors. The included radiological variables, T category, CRM involvement and size-based lymph node categories, were all of good to excellent interobserver agreement. Such a nomogram provided clinicians a consistent and reliable tool to further improve nodal staging in patients with rectal cancer.

There are limitations to the current study. The study design was retrospective and the fact of single institutional study may limit its applicability in other patients. Thus, additional validations in patients from multi-institutions are needed in the future. Moreover, the treatment in patients with cT3N0 rectal cancers was the area of greatest controversy. Because of the small number of cT3N0 cases in this series, we are not able to perform a reliable subgroup analysis in these patients. These will be conducted in our future investigation.

## CONCLUSIONS

By incorporating preoperative clinicpathological variables and magnetic resonance imaging features, we established a nomogram that improved the diagnostic accuracy and remarkably minimized the interobserver disagreement in diagnosing lymph nodes metastases in rectal cancers.

## MATERIALS AND METHODS

### Patients

All patients with rectal cancer were collected from a single institution, Fudan University Shanghai Cancer Center, with a high volume of rectal cancer patients each year. To determine the accurate status of lymph nodes metastases, all patients selected in our series underwent preoperative high resolution MRI scanning and then followed by immediate surgeries for resection the primary tumor under the principle of total mesorectal excision in our institution. Patients were excluded for following reasons: lack of preoperative high-resolution MRI staging, undergoing preoperative treatment (chemotherapy, CRT or radiotherapy), distant metastases and unresectable primary tumors.

Between January 2005 and December 2014, a total of 411 patients with rectal cancer were included in the current study. All patients were rectal adenocarcinoma located within 12 cm from anal verge. The main reasons for the “surgery first” strategy included MRI-staged early disease, patient's refusal of preoperative CRT, old age, doctor's preference, financial considerations and local insurance issues. All patients in our series underwent resection of the primary tumors with the principle of total mesorectal excision. All visible resected specimens were embedded in paraffin for 24 hours and examined histologically with hematoxylin-eosin (HE) staining. The extent of local tumor spread in each slice was assessed and the total number of histological detected lymph node was recorded. According to the histological report in our hospital database, the tumor staging was restaged using the 6th edition of TNM (tumor, node and metastasis) system [11]. The lymph node status was graded as: N0, no lymph node metastasis; N1, one to three lymph node metastasis; N2, four or more lymph node metastasis.

This study was approved by the Fudan University Shanghai Cancer Center Institutional Ethics Committee.

### MRI imaging

High-resolution scan parameters in preoperative MRI were used for each patient. The T2-weighted fast spin echo sequence with a thin 2-mm section was mainly used for preoperative assessment. Images were made in the sagittal, coronal and axial plane. The cranial border of the field of view was L5, the caudal border was below the anal canal. No intrarectal coli or contrast was used. Intravenous Gadolinium-enhanced scanning was used for most of the patients, but the enhancement of lymph nodes was not routinely assessed for each patient in this study.

The variables assessed by MRI included primary tumor and local or regional lymph nodes. In the training group, the assessment of primary tumor included cT category, CRM involvement, tumor spectrum within the bowel circle, tumor location related to peritoneal reflex; the assessment of lymph nodes included the number of detected nodes, sizes, irregularity of nodes' border and uniformity of signal intensity within the nodes. We recorded the total number of lymph nodes and the number of lymph nodes by different node sizes (< 5 mm, 5 mm–10 mm, > 10 mm). In the validation group, the variables used in the predictive nomogram (developed from the training data) were assessed.

All MRI images were assessed by two independent observers: Doc1 (TT) and Doc2 (LHX) with 5 and 15 years of experience in MRI imaging, respectively. Each observer was asked to make a diagnosis of N category (N+/N−) for each patient, and a consensus N category for each disagreed case was determined after discussion. We refer to the studies by Brown et al. and Kim et al. [19, 20] for the criteria for lymph node metastasis. The interobserver agreement was measured between the two observers according to their original diagnosis of N category.

### Statistics

The clinicopathological characteristics between the training group and the validation group were analyzed by chi-square test (age was dichotomized). The sensitivity, specificity, PPV and NPV of conventional MRI-assessed cN stage were calculated. Weighted kappa values were calculated to evaluate the interobserver agreement in cT stage and cN stage categorization, respectively [31].

### Statistical model development

To develop a nomogram with independent data to validate, of all the 411 patients, 288 patients, treated between January 2005 and December 2012, were assigned to the training group; and the other 123 patients, treated between January 2013 and December 2014, were assigned to the validation group.

In the training data, univariate logistic regression was performed for each potential predictive variable to assess its association with the pathological lymph node metastasis (N+/N−). The variables with univariate *p*-value < 0.1 were considered as candidate predictors in the multivariate logistic model. The final nomogram derived from the training data was selected by the stepwise multivariate logistic regression with post-hoc AUC examination.

### Model validation

The predictive nomogram for N category was evaluated in both the training group and the validation group. The predicted probability for pathological lymph node metastasis (pN+) from the nomogram was compared with the actual lymph node metastasis status (determined post-surgery by pathologists) for each patient. The ROC curves were plotted with AUCs calculated.

All the statistical analyses were performed using R 3.1. A *p* < 0.05 was considered statistically significant.
